# A mosquito AgTRIO mRNA vaccine contributes to immunity against malaria

**DOI:** 10.1038/s41541-023-00679-x

**Published:** 2023-06-07

**Authors:** Yu-Min Chuang, Mohamad-Gabriel Alameh, Selma Abouneameh, Hamidah Raduwan, Michel Ledizet, Drew Weissman, Erol Fikrig

**Affiliations:** 1 Section of Infectious Diseases, Department of Internal Medicine, Yale University School of Medicine, New Haven, CT USA; 2 Institute for RNA Innovation and Department of Medicine, University of Pennsylvania, Philadelphia, PA USA; 3 L2 Diagnostics, LLC, New Haven, CT USA

**Keywords:** Malaria, RNA vaccines, RNA vaccines

## Abstract

Malaria begins when an infected mosquito injects saliva containing *Plasmodium* sporozoites into the skin of a vertebrate host. To prevent malaria, vaccination is the most effective strategy and there is an urgent need for new strategies to enhance current pathogen-based vaccines. Active or passive immunization against a mosquito saliva protein, AgTRIO, contributes to protection against *Plasmodium* infection of mice. In this study, we generated an AgTRIO mRNA-lipid nanoparticle (LNP) and assessed its potential usefulness as a vaccine against malaria. Immunization of mice with an AgTRIO mRNA-LNP generated a robust humoral response, including AgTRIO IgG2a isotype antibodies that have been associated with protection. AgTRIO mRNA-LNP immunized mice exposed to *Plasmodium berghei*-infected mosquitoes had markedly reduced initial *Plasmodium* hepatic infection levels and increased survival compared to control mice. In addition, as the humoral response to AgTRIO waned over 6 months, additional mosquito bites boosted the AgTRIO IgG titers, including IgG1 and IgG2a isotypes, which offers a unique advantage compared to pathogen-based vaccines. These data will aid in the generation of future malaria vaccines that may include both pathogen and vector antigens.

## Introduction

Human malaria can be caused by at least 5 different species of *Plasmodium*^1^. To prevent infection, vaccination is the most effective strategy. However, no highly effective malaria vaccine has been developed to date^2–4^. Several vaccine candidates are in clinical trials. One of the most advanced vaccines—just approved by the WHO in 2021—is a pathogen-based vaccine, RTS,S. RTS,S targets the *Plasmodium falciparum* circumsporozoite protein (PfCSP) in conjunction with immunostimulatory epitopes of the hepatitis B surface antigen and AS01 adjuvant^5^. The protection efficacy is moderate with a 30–50% reduction in infection in clinical trials, and the protection wanes over time^5–8^. In addition, the protection is limited to *P. falciparum* and there is still a risk of infection by other *Plasmodium* species—two weaknesses that limit the possibility of malaria eradication^9^. Furthermore, the widespread emergence of drug resistance in malaria parasites and insecticide resistance in mosquito vectors makes it imperative to develop strategies to identify vaccine targets that synergize with CSP, or that work independently of CSP.

Malaria is transmitted when an infected female *Anopheles* mosquito takes a bloodmeal and injects *Plasmodium* sporozoites along with saliva into the skin of the vertebrate host^10,11^. Following a mosquito bite, sporozoites travel within blood vessels to the liver, where they invade hepatocytes and establish infection^12^. Mosquito saliva contains biologically active molecules, which modulate the host immune and hemostatic responses^13–20^, and may also influence *Plasmodium* infection. There are reports of intimate interactions between proteins in the salivary glands of *Anopheles* mosquitoes and *Plasmodium* prior to, and after, migration of *Plasmodium* out of the mosquito^21–24^. It has also been shown that hyperimmune sera against mosquito salivary gland extracts may contain high titer antibodies against components in saliva that are not normally antigenic during a natural mosquito bite, and that such sera may provide some protection against *Plasmodium* transmission^23^. Specifically, we have identified at least one antigen, AgTRIO from *Anopheles gambiae* saliva, that elicits antibodies that decrease malaria infection in both a traditional mouse model and a humanized mouse model of malaria^23^. AgTRIO antibodies also offer synergistic protection when combined with a CSP monoclonal antibody^23^. These studies suggest that immunization against AgTRIO may contribute to protection against *Plasmodium* infection and could be useful in combination with pathogen immunogens.

mRNA vaccines are an important technological advance to prevent infection diseases, as demonstrated by the SARS-CoV2 pandemic^25,26^. Several studies using mRNA vaccines targeting PfCSP proved its usefulness, albeit without complete protection, in murine models of *Plasmodium* infection^27–29^. Furthermore, targeting of several antigens is readily feasible using mRNA vaccines^29–31^, which can potentially generate a more potent antimalarial response to enhance a PfCSP-based vaccine. We have produced a lipid nanoparticle (LNP) containing an mRNA encoding the mosquito saliva protein AgTRIO. Immunization of C57BL/6 mice with the AgTRIO mRNA-LNP vaccine decreased the initial *Plasmodium* burden in the liver and subsequent parasitemia, following exposure to *Plasmodium*-infected mosquitoes. Furthermore, the IgG response against AgTRIO can be boosted by mosquito bites, which could potentially solve an issue related to the duration of protection seen in pathogen-based vaccines. Based on these data, an AgTRIO mRNA-LNP vaccine may be useful as part of a multiantigen vaccine approach against malaria.

## Results

### AgTRIO mRNA-LNP vaccine generates AgTRIO-specific antibodies in mice

**Fig. 1:**
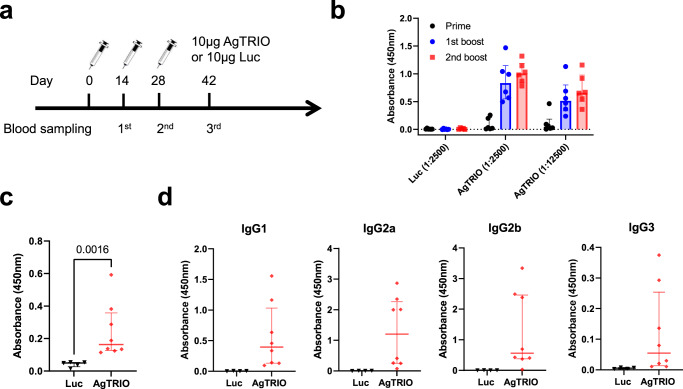
AgTRIO mRNA-LNP immunization generates a robust IgG response against recombinant AgTRIO and salivary gland extracts. **a** Experiment scheme showing groups of C57BL/6 female mice injected with 10 μg of AgTRIO mRNA-LNP or control mRNA (Luc mRNA-LNP), and boosted twice, at two-week intervals. **b** Two weeks after each immunization, mice were bled. 1:2,500 and 1:12,500 dilutions of sera were examined for AgTRIO-specific IgG antibodies by ELISA using recombinant AgTRIO as the antigen. **c** 1:2,500 dilution of sera collected after the final were examined for AgTRIO-specific IgG antibodies against salivary gland extracts by ELISA. (Median ± IQR, *p* < 0.05 using the Mann Whitney U-test) **d** 1:2,500 dilution of sera were used to determine AgTRIO-specific IgG1, IgG2a and IgG2b. 1:500 dilution of sera were examined for AgTRIO specific IgG3 antibodies.

To determine if AgTRIO mRNA-LNP can generate AgTRIO specific antibodies, C57BL/6 mice were immunized three times with 10 μg of AgTRIO mRNA-LNP or control, Luciferase (Luc) mRNA-LNP (Fig. 1a). Two weeks after each injection, blood was collected and analyzed by an enzyme-linked immunosorbent assay (ELISA) using a purified *E. coli* derived AgTRIO (Fig. 1b). Immunization with AgTRIO mRNA-LNP generated significant IgG responses after the first booster immunization. To determine if immunization resulted in antibodies that recognized native AgTRIO, we collected salivary gland extracts (SGE) from female *Anopheles gambiae* and then coated plates with SGE for ELISA. Immunization with AgTRIO mRNA-LNP generated significant antibodies against SGE (Fig. 1c). In our recent study, we found that the IgG subclass of AgTRIO monoclonal antibody (mAb) may influence the protective effect against *Plasmodium* infection^32^. In particular, a murine IgG2a mAb against AgTRIO offered the most potent protection^32^. By ELISA, we found that the AgTRIO mRNA-LNP generated diverse antibody isotypes against AgTRIO, including IgG2a antibodies (Fig. 1d).

### Immunization with AgTRIO mRNA-LNP alters mosquito-borne *Plasmodium* infection in mice

**Fig. 2:**
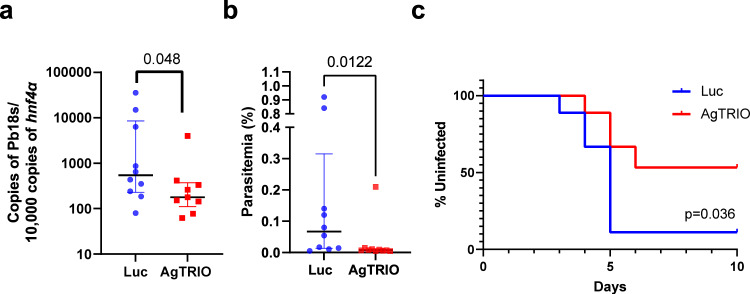
Immunization with AgTRIO mRNA-LNP reduces the initial Plasmodium berghei infection level and offers protection in mice. C57BL/6 mice were immunized with 10 µg of AgTRIO mRNA-LNP or control mRNA (Luc) three times. 3 weeks after last boost, the immunized mice were exposed to 3 *P. berghei*-infected mosquitoes**. a** After 40 h, livers were dissected, and the *Plasmodium* infection level was determined by RT-PCR. (Median ± IQR, *p* < 0.05 using the Mann Whitney U-test). **b** In a separate experiment with mice vaccinated in the same way and exposed to infected mosquito bites, the parasitemia levels were determined at day 5 post infection. (Median ± IQR, *p* < 0.05 using the Mann Whitney U-test). **c** Kaplan–Meier survival curves were used to present the percent of uninfected mice. Infection was determined as 0.01% of parasitemia by flow cytometry. *p* = 0.036 by Log rank test between the two groups.

In our previous study, active immunization with AgTRIO or passive transfer with AgTRIO antisera offered partial protection against mosquito-borne *Plasmodium* infection in mice^23^.To determine whether immunization with AgTRIO mRNA-LNP can influence *Plasmodium* infection, we immunized C57BL/6 mice with 10 μg AgTRIO mRNA-LNP three times at two-week intervals. The control group received 10 μg Luc RNA-LNP. Three weeks after the last immunization, we challenged the mice using 3 *P. berghei*-infected *A gambiae* mosquitoes each. Mice immunized with AgTRIO mRNA-LNP had median hepatic levels of *P. berghei* reduced by 68% compared to those immunized with Luc mRNA-LNP (Fig. 2a, *p* = 0.048). To further examine the effects of this AgTRIO mRNA-LNP on the development of the later stages of malaria infection, such as parasitemia, we immunized an additional group of mice with either 10 μg AgTRIO mRNA-LNP or Luc mRNA-LNP thrice and then exposed the animals to three *P. berghei*-infected mosquitoes. 5 days after exposure to infected mosquito bites, there was significantly less parasitemia in the group received the AgTRIO mRNA-LNP vaccine (Fig. 2b, *p* = 0.012). Then we used Kaplan-Meier survival curves to demonstrate the kinetics of 0.01% parasitemia as the evidence of infection (Fig. 2c). Consistent with liver burden and Day 5 parasitemia results, there were less infected mice in the group given the AgTRIO mRNA-LNP vaccine (*p* = 0.036 by Log rank test). All these data demonstrate that immunization with AgTRIO mRNA-LNP can markedly reduce the hepatic *Plasmodium* burden in the murine model and contribute to protection against malaria.

### Humoral responses elicited by the AgTRIO mRNA-LNP can be enhanced by mosquito bites

**Fig. 3:**
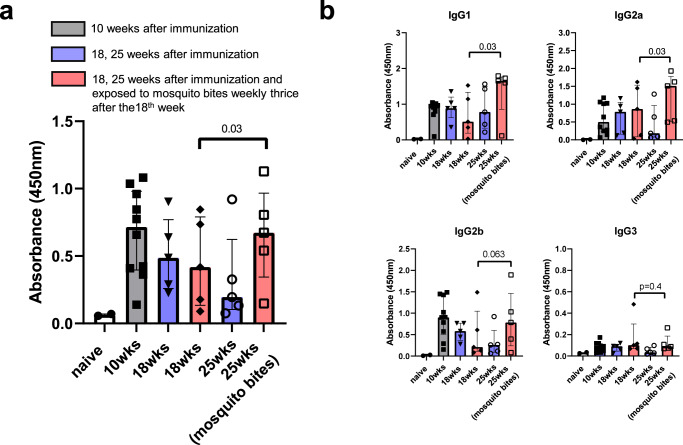
Mosquito bites can maintain AgTRIO-specific IgG, including IgG2a antibodies, after AgTRIO mRNA-LNP immunization. A group of mice was injected with 10 μg of AgTRIO mRNA-LNP or control mRNA (Gluc mRNA-LNP) and boosted twice over 4 weeks. Sera were collected at 10, 18, and 25 weeks after the beginning of the experiments. **a** 1:2,500 dilution of sera were examined for total AgTRIO-specific IgG antibodies by ELISA**. b** 1:2,500 dilution of sera were used to determine AgTRIO specific IgG1, IgG2a, and IgG2b. 1:500 dilution of sera were examined for AgTRIO specific IgG3 antibodies. (Median ± IQR, *p* < 0.05 using the Wilcoxon matched-pairs signed rank test). Red bar: exposed to 10 mosquitoes weekly after 18 weeks for three times. Blue bar: non-exposure.

Since AgTRIO is highly expressed in mosquito saliva^23^, we then examined whether the declining IgG responses that naturally occurs following any vaccination can—in the case of AgTRIO—be boosted by mosquito bites. We immunized C57BL/6 mice with three doses of AgTRIO mRNA-LNP and determined IgG titers at regular intervals. By 18 weeks after the beginning of the experiments (14 weeks after the final booster immunization), the IgG titers against AgTRIO (median: 0.45) had decreased by approximately 40% compared to 10 weeks after the beginning of the experiments (median: 0.716, Fig. 3a). Then, one group of mice was exposed to 10 uninfected mosquito bites weekly for 3 weeks and the control group of mice was not exposed to AgTRIO. When feeding on mice, there were no significant adverse effects noted during repeated mosquito bites. The anti-AgTRIO IgG concentration increased markedly in the group exposed to mosquito bites while the IgG levels continued to decrease in the control group (Fig. 3a, *p* = 0.03 by the Wilcoxon matched-pairs signed rank test). Next, we determined which IgG subclasses of AgTRIO specific antibody were boosted by mosquito bites. By ELISA, AgTRIO-specific IgG1 and IgG2a were significantly increased after mosquito bites (Fig. 3b). These results show that mosquito bites can maintain the IgG response against AgTRIO following immunization, including IgG isotypes that have been associated with protection^32^.

## Discussion

When probing for blood meal, mosquitoes secrete saliva to facilitate feeding, and sporozoites are simultaneously released into the skin^10,11^. Therefore, components of saliva can directly or indirectly affect the transmission of *Plasmodium* at the bite site and are potential targets for disease prevention^23,33^. In our previous studies, we demonstrated that passive or active immunization against one of the salivary proteins, AgTRIO, offers partial protection against *P. berghei* and *P. falciparum* transmitted by *A. gambiae* or *A. stephensi* mosquito bites^23^. In addition, we demonstrated that AgTRIO is highly secreted in mosquito saliva, and mosquitoes in which AgTRIO has been silenced by RNAi do not efficiently transmit sporozoites to murine skin compared with control mosquitoes^22,23^. This makes AgTRIO a suitable candidate for inclusion in a multi-component vaccine. In this study, we generated an AgTRIO mRNA-LNP that induces AgTRIO-specific humoral responses, including IgG2a isotype antibodies that have been associated with protection^32^. The AgTRIO mRNA-LNP significantly reduced *Plasmodium* infection in mice. Our finding indicates that immunization against AgTRIO, using an mRNA approach, can contribute to protection against *Plasmodium* transmission from the arthropod to a vertebrate host.

In our previous study, mice immunized with AgTRIO protein in complete Freund’s adjuvant and then boosted with AgTRIO protein with incomplete Freund’s adjuvant had a 50% lower liver burden compared to the control group^23^. In the same study, 66% percent of the AgTRIO-immunized mice had detectable parasitemia compared to 95% of the control mice^23^. While Freund’s adjuvant is useful for demonstrating proof of principle as it is highly immunostimulatory, and it cannot be given to humans because of its toxic side effects. Using the AgTRIO mRNA approach, 44.4% (4/9) of mice that received AgTRIO mRNA-LNP reached a 0.01% parasitemia level after challenge compared to 88.8% (8/9) of the control group. In our previous study, an IgG2a AgTRIO monoclonal antibody offered substantial protection against *Plasmodium* infection in mice compared to the other isotype monoclonal antibodies, and the protection depended on the Fc region^32^. In our unpublished results, there were AgTRIO specific IgG1 and IgG2b responses after immunization with AgTRIO protein and Alum adjuvant, but there was no significant IgG2a response. AgTRIO-mRNA LNP provides a robust IgG isotype response, including the generation of IgG2a antibodies, which may offer more effective protection against *Plasmodium* infection. All these results demonstrate that the AgTRIO mRNA-LNP approach may offer advantages compared with protein-based vaccines.

In addition, following an infection or vaccination, waning of protective immune responses occurs over time and is the major concern. As an example, for most COVID-19 vaccines, the humoral response diminishes more that 50% after 6 months^34–38^. To maintain protective humoral responses, a boosting vaccine is required. Similar to other vaccines, PfCSP-based vaccines exhibit waning protection over time^6,7^. While the humoral response towards AgTRIO declined after 6 months of immunization, antibodies could be boosted by mosquito bites. On the other hand, there is no significant humoral immune response against AgTRIO after repeat mosquito bites in either human or mice^23^, which indicates the boosting effect only occurs following priming by active immunization with AgTRIO. There were no significant adverse effects, including redness, swelling or an allergic reaction, noted during repeated mosquito bites after immunization with AgTRIO mRNA-LNP vaccine in our study. However, it is important to examine safety directly in human as results in mice do not always correlate directly with humans. This naturally acquired boosting effect gives AgTRIO-based immunization a unique advantage over traditional pathogen-based vaccine. In malaria endemic areas, persistent exposure to the vector could readily increase and maintain AgTRIO antibodies in AgTRIO mRNA-LNP vaccinated individuals. Overall, our study demonstrates that an mRNA vaccine strategy targeting a mosquito saliva protein can aid in the development of the next generation of vaccines against malaria, and may be used in conjunction with traditional, *Plasmodium*-based vaccines.

## Methods

### Animals

*A. gambiae* (4arr strain) mosquitoes were raised at 27 °C, 80% humidity, under a 12/12-h light/dark cycle and maintained with 10% sucrose under standard laboratory conditions in the insectary at Yale University. Swiss Webster and C57BL/6 mice were purchased from Charles River Laboratories. All animal experiment protocols were approved by the Yale University Institutional Animal Care & Use Committee (Protocol Number: 2022–07941). All *Plasmodium* infections were performed in biosafety level 2 animal facilities. All animal experiments followed the Guide for the Care and Use of Laboratory Animals by the National Research Council. In all experiments, mice were housed and cared for in the Association for Assessment and Accreditation of Laboratory Animal Care International (AAALAC)-accredited animal facilities in Yale University. For retro-orbital blood collection or mosquito feeding, mice were anesthetized with intraperitoneal injection of Ketamine/Xylazine (100 mg/10 mg per kg body weight). When the whole experiments were finished, mice were euthanized using a CO2 chamber in a manner consistent with AVMA guidelines for euthanasia. Following death, mice were subject to cervical dislocation as a secondary means to ensure death.

### AgTRIO LNP mRNA synthesis

AgTRIO (AGAP001374) without the signal peptide were codon optimized between the 22^th^ and 389^th^ amino acids and then AgTRIO or luciferase (control) mRNAs were transcribed to contain 101 nucleotide-long poly(A) tails. N-1-methylpseudouridine (m1Ψ-5′)-triphosphate instead of UTP was used to generate modified nucleoside-containing mRNA. Capping of the in vitro transcribed mRNAs was performed co-transcriptionally using the trinucleotide cap1 analog, CleanCap. mRNA was purified by cellulose purification^39^. The mRNA was then encapsulated in LNPs using an aqueous solution of mRNA at acidic pH 4.0 and mixed with a solution of lipids^40,41^, consisting of an ionizable cationic lipid/phosphatidylcholine/cholesterol/PEG-lipid (50:10:38.5:1.5 mol/mol)^28–31^. For encapsulation, RNA was mixed with the lipids at a ratio of ∼0.05 (wt/wt). The LNP had a diameter of ∼80 nm as measured by dynamic light scattering using a Zetasizer Nano ZS (Malvern Instruments Ltd, Malvern, UK) instrument, and is stored at −80 °C.

### Active immunization

Five-week-old female C57BL/6 mice were immunized with either 10 µg of AgTRIO mRNA-LNP or control (Luc mRNA-LNP) and then given boosts with the same amount of vaccine 2 and 4 weeks later. Two weeks after each immunization, serum was collected from each mouse, and the IgG responses were tested using an ELISA to confirm antigen-specific antibodies. To determine if mosquito bite can boost the IgG, after the AgTRIO IgG titers were decayed, we let 10 uninfected mosquitoes feed on the mice. The control mice were not exposed to mosquitoes.

### Plasmodium burden

The burden of *Plasmodium* in livers after sporozoite infection was determined by assessing the expression level of *P. berghei 18* *S rRNA*, normalized to *M. musculus* hepatocyte nuclear factor 4 alpha, *hnf4α*, which has been used as housekeeping gene for liver tissue^23,33^. These data were presented as the copy number of the target gene per 10,000 copies of the housekeeping gene. TRIzol Reagent (Thermo Fisher Scientific) was used to purify total RNA from murine livers. All extractions followed the manufacturer’s protocols. The iScript RT-qPCR kit (Bio-Rad) was used to generate cDNA from RNA. Using iTaq SYBR Green Supermix (Bio-Rad), real time PCR was performed on a CFX96 real time platform (Bio-Rad). PCR involved an initial denaturation at 95 °C for 2 min, 50 cycles of 15 s at 95 °C, 15 s at 60 °C, and 20 s at 72 °C. Fluorescence readings were taken at 72 °C after each cycle. At the end of each reaction, a melting curve (60–95 °C) was checked to confirm the identity of the PCR product. The primers used for the expression of sporozoite genes are listed in Table 1. To assess parasitemia, 20 μl of blood was collected from all mice by retro-orbital bleeding. The percentage of parasitemia were determined by comparing the GFP-positive RBCs to the total RBC count by flow cytometry (CytoFLEX, ThermoFisher).

**Table 1:** Primers used for protein expression and RT-PCR.

	Primer Name	Sequence 5′-3′	Reference
**RT-PCR for** ***P. berghei***			
Pb 18s	*Pb18* *s* F	AAGCATTAAATAAAGCGAATACATCCTTAC	^22^
	*Pb18* *s* R	GGAGATTGGTTTTGACGTTTATGTG	
**RT-PCR for mice**			
HNF4α	*HNF4α* F	TCAAGGATGAAGAGCTTGCC	^23^
	*HNF4α* R	ACGTGTCTGATGTGATCTGC	

### AgTRIO protein expression and purification

The *AgTRIO* genetic sequence without the region corresponding to the signal peptide has been cloned to the bacterial expression vector, pET21b (GE Healthcare)^23^. The *AgTRIO*-expressing plasmid will be transformed into BL21 chemically competent cells (Thermo Fisher Scientific). Expression of the AgTRIO protein will be induced with 0.1 mM IPTG at 17 °C for 24 h. The cells will be sonicated in PBS with complete EDTA-free Protease Inhibitors tablets (Roche). Soluble AgTRIO will be purified from the supernatant by a combination of sizing, ion-exchange and affinity chromatography. The expression of recombinant AgTRIO will be confirmed by immunoblots, which will be probed with the anti-AgTRIO serum raised against AgTRIO from our previous study^23^. Protein purity will be determined by SDS-PAGE and the concentration will be determined by the BCA Protein assay kit (Pierce, IL).

### Enzyme-linked immunosorbent assays (ELISA)

Antigen-specific antibody responses were measured by ELISA^42,43^. To prepare the salivary gland extract (SGE), 20 salivary glands were collected in 100 μl PBS and the concentration of SGE protein was 0.2 mg/ml. The 96-well microplates were coated with purified AgTRIO (1 µg/ml) or SGE overnight. After blocking with a blocking buffer (PBS, 0.05% tween-20, and 1% bovine serum albumin), different dilution of mouse antisera were diluted in PBS and added to the wells and incubated at room temperature for 2 hours. After washing with washing buffer (PBS, 0.1% tween-20), horseradish peroxidase-conjugated goat anti-mouse antibody (Invitrogen, Cat. No. 62–6520) with 1:10000 dilution was used to detect total IgG. For the detection of IgG subclasses, horseradish peroxidase-conjugated goat anti-mouse IgG1, IgG2a, IgG2b, or IgG3 heavy chain antibodies (Abcam, Cat. No. ab97240, ab97245, ab97250, ab97260) with 1:10,000 dilution was used to detect specific IgG subclass.

### P. berghei infection

*P. berghei* (ANKA GFPcon, ATCC) were maintained by serial passage in 6–8 week old female Swiss Webster or C57BL/6 mice as previously described^23^. Briefly, Swiss Webster or C57BL/6 mice were challenged with *P. berghei*-infected red blood cells by intraperitoneal injection. *A. gambiae* mosquitoes then took a blood meal from the infected mice, when the parasitemia was approximately 5%. 17–24 days after *P. berghei* infection, the mosquitoes were sorted using the fluorescent signal of the salivary glands.

### Statistical analysis

Data from at least three biological replicates were used to calculate medians for graphing purposes. Statistical analyses employed the Mann-Whitney test for unrelated groups and the one-sided Wilcoxon matched-pairs signed rank test for the same group with serial sampling to determine if the mosquito bites increased AgTRIO specific IgGs by ELISA. The difference and the data were presented as median with interquartile range (IQR). A *p-*value of <0.05 was considered statistically significant. The analysis, graphs, and statistics of all data were performed using Prism 9.0 software (GraphPad Software).

### Reporting summary

Further information on research design is available in the Nature Research Reporting Summary linked to this article.

## Supplementary information

REPORTING SUMMARY

## Data Availability

All data associated with this study are present in the paper. Requests for resources, data, and reagents should be directed to the corresponding author, Dr. Y.-M.C. (yu-min.chuang@yale.edu). All unique reagents described in this study are available upon request to the corresponding author with a completed Materials Transfer Agreement.
