# Supplementary material for: A mosquito AgTRIO mRNA vaccine contributes to immunity against malaria
Source: NPJ Vaccines. 2023 Jun 7;8:88. doi: 10.1038/s41541-023-00679-x (PMC10244833; doi:10.1038/s41541-023-00679-x)
Supplement: Supplementary file 1 — REPORTING SUMMARY [file 41541_2023_679_MOESM1_ESM.pdf]

nature portfolio

Corresponding author(s): Yu-Min Chuang

Last updated by author(s): May 8, 2023

Reporting Summary

Nature Portfolio wishes to improve the reproducibility of the work that we publish. This form provides structure for consistency and transparency in reporting. For further information on Nature Portfolio policies, see our Editorial Policies and the Editorial Policy Checklist.

Statistics

For all statistical analyses, confirm that the following items are present in the figure legend, table legend, main text, or Methods section.

n/a	Confirmed	
☐	☒	The exact sample size (n) for each experimental group/condition, given as a discrete number and unit of measurement
☐	☒	A statement on whether measurements were taken from distinct samples or whether the same sample was measured repeatedly
☐	☒	The statistical test(s) used AND whether they are one- or two-sided<br>*Only common tests should be described solely by name; describe more complex techniques in the Methods section.*
☐	☒	A description of all covariates tested
☐	☒	A description of any assumptions or corrections, such as tests of normality and adjustment for multiple comparisons
☐	☒	A full description of the statistical parameters including central tendency (e.g. means) or other basic estimates (e.g. regression coefficient) AND variation (e.g. standard deviation) or associated estimates of uncertainty (e.g. confidence intervals)
☐	☒	For null hypothesis testing, the test statistic (e.g. F, t, r) with confidence intervals, effect sizes, degrees of freedom and P value noted<br>*Give P values as exact values whenever suitable.*
☒	☐	For Bayesian analysis, information on the choice of priors and Markov chain Monte Carlo settings
☒	☐	For hierarchical and complex designs, identification of the appropriate level for tests and full reporting of outcomes
☒	☐	Estimates of effect sizes (e.g. Cohen's d, Pearson's r), indicating how they were calculated

Our web collection on statistics for biologists contains articles on many of the points above.

Software and code

Policy information about availability of computer code

Data collection	No software was used.
Data analysis	All data were performed using Prism 9.0 software (GraphPad Software).

For manuscripts utilizing custom algorithms or software that are central to the research but not yet described in published literature, software must be made available to editors and reviewers. We strongly encourage code deposition in a community repository (e.g. GitHub). See the Nature Portfolio guidelines for submitting code & software for further information.

Data

Policy information about availability of data

All manuscripts must include a data availability statement. This statement should provide the following information, where applicable:

- Accession codes, unique identifiers, or web links for publicly available datasets
- A description of any restrictions on data availability
- For clinical datasets or third party data, please ensure that the statement adheres to our policy

All data associated with this study are present in the paper. Request for resources, data and reagents should be directed to the corresponding author, Dr. Yu-Min Chuang (yu-min.chuang@yale.edu). All unique reagents described in this study are available upon request to the corresponding author with a completed Materials Transfer Agreement.

Field-specific reporting

Please select the one below that is the best fit for your research. If you are not sure, read the appropriate sections before making your selection.

☒ Life sciences ☐ Behavioural & social sciences ☐ Ecological, evolutionary & environmental sciences

For a reference copy of the document with all sections, see nature.com/documents/nr-reporting-summary-flat.pdf

Life sciences study design

All studies must disclose on these points even when the disclosure is negative.

Sample size	Based on our previous studies, the sample size between 5-15 mice is adequate to see the protection effects of vaccine targeting mosquito saliva proteins.
Data exclusions	No data were excluded.
Replication	Data from at least three biological replicates were used to calculate medians for graphing purposes.
Randomization	When immunized mice exposed to infected mosquitoes, the selection of mosquitoes were randomized.
Blinding	During mosquito challenge experiments, it was not applicable for blinding. But, when we interpreted the results, it was unbiased and could be affected by interpretors.

Reporting for specific materials, systems and methods

We require information from authors about some types of materials, experimental systems and methods used in many studies. Here, indicate whether each material, system or method listed is relevant to your study. If you are not sure if a list item applies to your research, read the appropriate section before selecting a response.

Materials & experimental systems

n/a	Involved in the study	
☐	☒	Antibodies
☒	☐	Eukaryotic cell lines
☒	☐	Palaeontology and archaeology
☐	☒	Animals and other organisms
☒	☐	Human research participants
☒	☐	Clinical data
☒	☐	Dual use research of concern

Methods

n/a	Involved in the study	
☒	☐	ChIP-seq
☒	☐	Flow cytometry
☒	☐	MRI-based neuroimaging

Antibodies

Antibodies used	HRP goat anti-mouse antibody (Invitrogen, # 31430, AB_228307) and HRP goat anti-mouse IgG1, IgG2a, IgG2b or IgG3 heavy chain antibodies (Abcam, ab97240, ab97245, ab97250, ab97260) were used to detect specific IgGs.
Validation	HRP goat anti-mouse antibody (Invitrogen, # 31430, AB_228307) are affinity-purified antibodies with well-characterized specificity for mouse immunoglobulins and are useful in the detection, sorting or purification of its specified target.<br>By immunoelectrophoresis and ELISA, HRP goat anti-mouse IgG1, IgG2a, IgG2b or IgG3 heavy chain antibodies (Abcam, ab97240, ab97245, ab97250, ab97260) react specifically with each mouse IgG subclass . Cross reactivity with other immunoglobulins is less than 2%.

Animals and other organisms

Policy information about studies involving animals; ARRIVE guidelines recommended for reporting animal research

Laboratory animals	Five-week-old female C57BL/6 mice were immunized in this stduy.
Wild animals	The study did not involved wild animals..
Field-collected samples	The study did not involved field-collected samples.
Ethics oversight	All animal experiment protocols were approved by the Yale University Institutional Animal Care & Use Committee (Protocol Number: 2022-07941).

Note that full information on the approval of the study protocol must also be provided in the manuscript.
